# Ontario Veterinary College

**Published:** 1897-01

**Authors:** 


### COMMENCEMENT EXERCISES.

### ONTARIO VETERINARY COLLEGE.

THE regular midwinter examination was held at the College on December 22d, when the following successful candidates obtained the diploma of the school: F. G. Atwood, Minertown, Conn.; A. McKay, Brock, Ottawa, Ontario; Eugene Elwood Burdick, Ashaway, Rhode Island; A. Edwin Dennis, Kinsale, Ontario; John P. Fitzgerald, Mount St. Louis, Ontario; Joseph Gregg, Little Britain, Ontario; Henry F. Hartnett, Brooklyn, N. Y.; Jeremiah J. Keleher, Pembroke, N. Y.; George H. Leslie, Ottawa, Ontario; David F. Luckey, Perryville, Missouri; A. R. Metcalfe, Van Kleek Hill, Ontario; G. H. Munro, Carluke, Ontario; Joseph Nelson, Bath, Ontario; Walter H. Orme, London, Ontario; James E. Smith, Webster, N. Y.; Joseph Tefler, Milton, Ontario; G. A. Wehr, Andreas, Penna.

---

The castration of Angora cats, where the long hair covers the scrotal sheath, makes necessary the close removal of the hair. A recent case operated upon at the Philadelphia Veterinary Sanitarium was sent home after twenty-four hours, apparently in good condition for the completion of the healing process. The suppuration of the parts became thoroughly matted with the hair, and proper retraction of the cords was hindered by becoming engaged in the lips of the scrotal incisions, and tension on the cords produced, giving rise to pain and discomfort. The thorough anointing of the parts and removal of the accumulated mass, with a reopening of the incisions, permitted the healing process to be completed without further incident.

